# Range extension of *Amolopshimalayanus* (Boulenger, 1888) (Anura, Ranidae), first record from China and first description of the juvenile of this species

**DOI:** 10.3897/BDJ.13.e148957

**Published:** 2025-03-14

**Authors:** JiShan Wang, Shuo Liu, Hengying Wang, Mo Wang, Dingqi Rao

**Affiliations:** 1 Southwest Survey and Planning Institute of National Forestry and Grassland Administration, Kunming, China Southwest Survey and Planning Institute of National Forestry and Grassland Administration Kunming China; 2 Yunnan Key Laboratory of Biodiversity Information, Kunming Institute of Zoology, Chinese Academy of Sciences, Kunming, China Yunnan Key Laboratory of Biodiversity Information, Kunming Institute of Zoology, Chinese Academy of Sciences Kunming China; 3 Kunming Natural History Museum of Zoology, Kunming Institute of Zoology, Chinese Academy of Sciences, Kunming, China Kunming Natural History Museum of Zoology, Kunming Institute of Zoology, Chinese Academy of Sciences Kunming China; 4 Key Laboratory of Biodiversity Conservation in Southwest China (State Forestry and Grassland Administration) / Yunnan Academy of Biodiversity, Southwest Forestry University, Kunming, China Key Laboratory of Biodiversity Conservation in Southwest China (State Forestry and Grassland Administration) / Yunnan Academy of Biodiversity, Southwest Forestry University Kunming China; 5 Kunming Institute of Zoology, Chinese Academy of Sciences, Kunming, China Kunming Institute of Zoology, Chinese Academy of Sciences Kunming China

**Keywords:** 16S rRNA, distribution, morphology, Yadong County, Xizang Autonomous Region

## Abstract

**Background:**

*Amolopshimalayanus* (Boulenger, 1888) is a poorly-known species which was originally described from north-eastern India over a hundred years ago. Currently, *A.himalayanus* is known only from India and Nepal and there is no reliable re-description or photos of this species reported.

**New information:**

We record *Amolopshimalayanus* (Boulenger, 1888) from China for the first time, based on one specimen collected from Yadong County, Xizang Autonomous Region, China. Although the specimen from China is a juvenile, however, phylogenetically, it was clustered with the syntype of *A.himalayanus* and the specimens of this species from Nepal with strong support and the genetic distance between the specimen from China and the syntype of *A.himalayanus* was only 0.7% in 16S gene sequences. We provide a description of the juvenile specimen and, in addition, we provide reliable photos of this species in life for the first time.

## Introduction

The genus *Amolops* Cope, 1865 is the most speciose genus in the family Ranidae (Frost 2025). This genus is widely distributed from Nepal, northern India, western and southern China to Malay Peninsula (Dever et al. 2012, Pham et al. 2019, Wu et al. 2020, Mahony et al. 2022, Tang et al. 2023, Li et al. 2024, Wu et al. 2024, Frost 2025). It currently includes 88 species, which can be subdivided into 10 species groups (Liu et al. 2025, Nguyen et al. 2025, Wu et al. 2025). In China, 60 species of *Amolops* have been recorded, 16 of which were found in Xizang Autonomous Region (Frost 2025, Huang et al. 2025, Liu et al. 2025).

*Amolopshimalayanus* (Boulenger, 1888) is a poorly-known species which was originally described from Darjeeling India over a hundred years ago and was later recorded in Nepal and Bhutan (Nidup et al. 2016, Limbu et al. 2020). However, Mahony et al. (2022) reviewed the species of *Amolops* in Bhutan and removed *A.himalayanus* from Bhutan’s amphibian checklist and confirmed that this species is present in Nepal. Currently, *A.himalayanus* is known only from north-eastern India and eastern Nepal (Frost 2025).

During our field survey in Xizang Autonomous Region, China, in 2017, a specimen of *Amolops* was collected from Yadong County. As this specimen is a juvenile, it cannot be accurately identified morphologically, we conducted molecular analysis for it and the result indicated that it is *A.himalayanus*. Therefore, we report the distribution of this species in China for the first time and provide a description of the specimen collected from China.

## Materials and methods

The field survey was carried out during the implementation of the General Survey on Forest Harmful Organisms project in Xizang, China. The frog specimen was collected as the certificate of forest pest’s natural enemy. After being photographed, the specimen was preserved in 75% ethanol and was deposited at Kunming Natural History Museum of Zoology, Kunming Institute of Zoology, Chinese Academy of Sciences (KIZ).

Measurements were taken with a digital caliper to the nearest 0.1 mm. The methodology of measurements followed Wu et al. (2024): SVL, snout-vent length, measured from the tip of the snout to the vent; HL, head length, measured from the tip of the snout to the angle of the jaw; HW, head width, measured at the widest point of the head; SL, snout length, measured from the tip of the snout to the anterior corner of the eye; INS, internasal space, measured between the nares; IOS, interorbital space, measured at the narrowest point between the eyelids; NED, nasal to eye distance, measured from the anterior corner of the eye to the centre of the nostril; UEW, upper eyelid width, measured at the maximal width of the upper eyelid; ED, eye diameter, measured between the anterior and posterior corners of the eye; TD, tympanum diameter, measured at the maximal diameter of the tympanum; LAHL, lower arm and hand length, measured from the elbow to the tip of the third finger; HND, hand length, measured from the proximal edge of the inner metacarpal tubercle to the tip of the third finger; LAD, lower arm diameter, measured at the maximal diameter of the lower arm; FEM, femoral length, measured from the cloaca to the knee; TIB, tibia length, measured from the knee to the heel; FTL, foot length, measured from the proximal end of the inner metatarsal tubercle to the tip of the fourth toe.

Total genomic DNA was extracted from liver tissue sample. A partial fragment of the mitochondrial 16S rRNA gene (16S) was amplified and sequenced using the primers 16Sar (5’-CGCCTGTTTAYCAAAAACAT-3’) and 16Sbr (5’-CCGGTYTGAACTCAGATCAYGT-3’) (Hedges 1994). The experimental protocols of amplification and sequencing followed Wu et al. (2024). The sequence was assembled using SeqMan in Lasergene 7.1 (Burland 2000). The new sequences have been deposited in GenBank and other sequences used in this study were obtained from GenBank (Table 1).

Sequences were aligned using MAFFT 7 (Katoh and Standley 2013) with default parameters. The best substitution models for Bayesian Inference (GTR+F+I+G4) and Maximum Likelihood phylogenetic analysis (GTR+F+R4) were selected using the Akaike Information Criterion in ModelFinder (Kalyaanamoorthy et al. 2017). The technical computation methods for Bayesian Inference and Maximum Likelihood analysis and genetic divergences calculation were the same as those in Liu et al. (2024).

## Taxon treatments

### Amolops himalayanus

(Boulenger, 1888)

0CB5657B-36E3-5D54-B55D-5F43CB3EA66D

#### Materials

- **Type status:** Other material. **Occurrence:** catalogNumber: KIZ 2017002; individualCount: 1; lifeStage: juvenile; occurrenceID: 43DC4C6E-ED61-558F-8083-2CF830E39168; **Taxon:** scientificName: *Amolopshimalayanus*; **Location:** country: China; stateProvince: Xizang; locality: Pangda Village, Xiayadong Township, Yadong County, Rikaze City; verbatimElevation: 1860 m; verbatimCoordinates: 27°15'13"N 89°1'16"E; **Event:** eventRemarks: collected by Hengying Wang on 27 July 2017; **Record Level:** basisOfRecord: preserved specime

#### Description of the specimen from China

**Figure 1.**
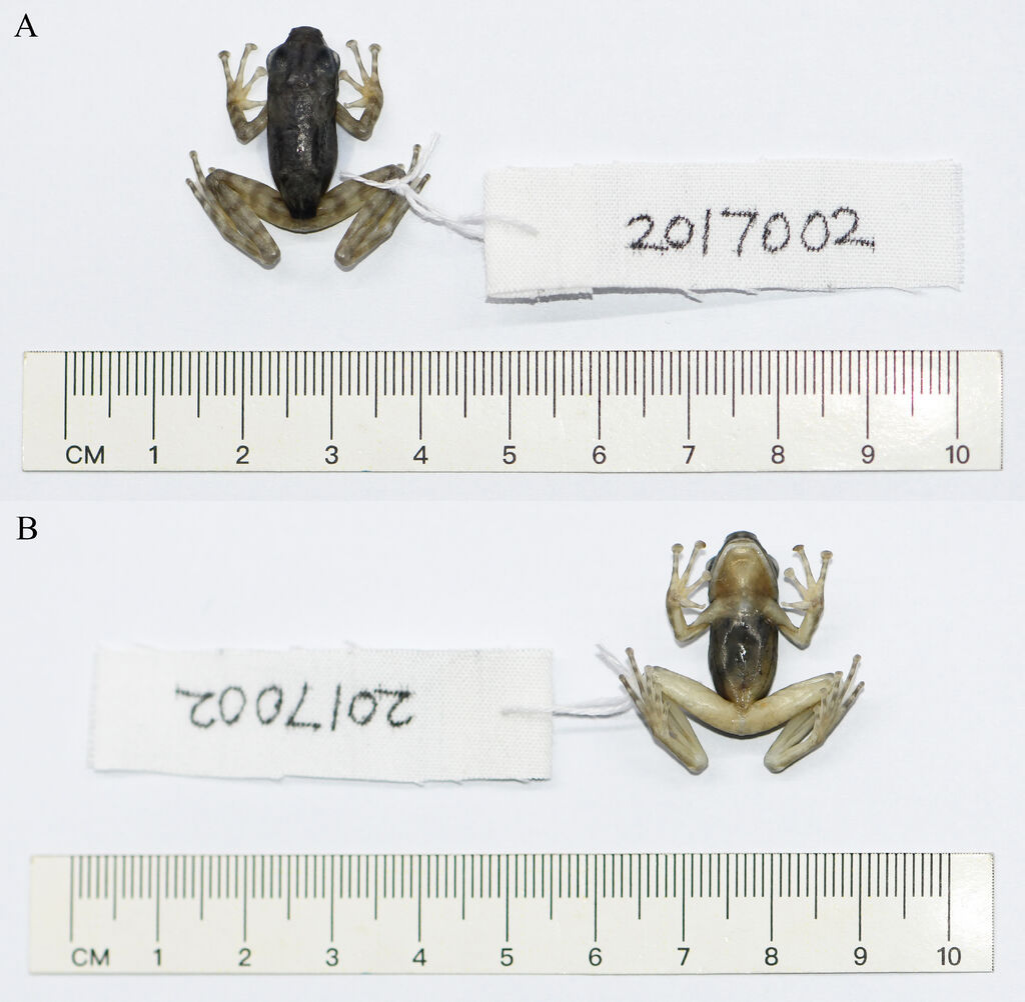
The specimen of *Amolopshimalayanus* from China in preservative. **A** Dorsal view; **B** ventral view.

Morphological measurements of the specimen (Fig. 1) are provided in Table 2. SVL 22.9 mm; head moderate long (HL/SVL 0.38), slightly longer than wide (HL/HW 1.08); snout moderate long (SL/SVL 0.16), projecting beyond lower jaw; canthus rostralis distinct; loreal region slightly concave; distance from nostril to snout tip slightly smaller than distance from eye to nostril; internarial distance slightly greater than interorbital distance (INS/IOS 1.07); upper eyelid width almost equal to interorbital distance (UEW/IOS 1.05); pupil oval, horizontal; tympanum distinct, small (TD/ED 0.27); tympanum to eye distance greater than tympanum diameter; pineal spot present, indistinct.

Fore-limb relatively long; relative length of fingers III > IV > II > I; tips of outer three fingers expanded into discs, circummarginal grooves present on tips of outer three fingers, absent on first finger; webbing between fingers absent; subarticular tubercles distinct, oval, formula 1, 1, 2, 2; supernumerary tubercles absent; metacarpal tubercles indistinct.

Hind-limb moderate long; tibia almost equal to femoral length (TIB/FEM 1.03); relative length of toes IV > V > III > II > I; all toe tips expanded into discs with circummarginal grooves; webbing between toes deeply incurved; subarticular tubercles distinct, oval, formula 1, 1, 2, 3, 2; supernumerary tubercles absent; inner metatarsal tubercle elongated; outer metatarsal tubercle absent.

Dorsal and lateral surfaces of head and body smooth; dorsal surface of fore-limb smooth, dorsal surface of hind-limb with many small tubercles; supratympanic fold present; discontinuous glandular dorsolateral fold from rear of eye to near vent; ventral surface smooth.

#### Colouration in life

**Figure 2.**
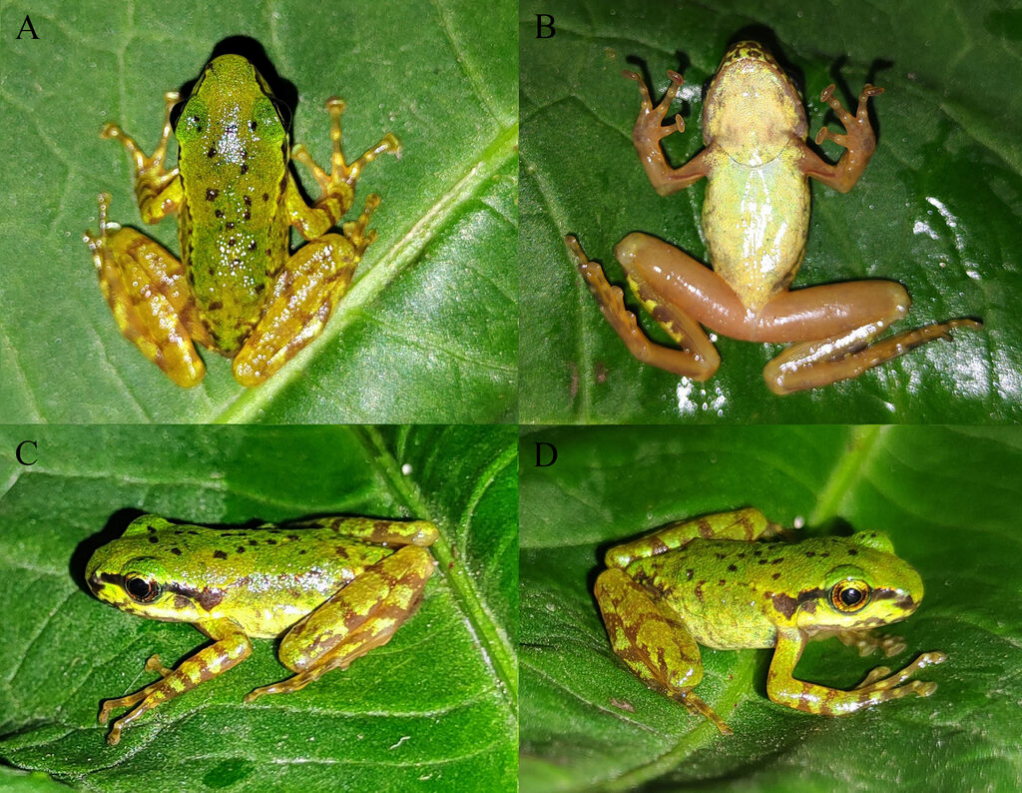
The specimen of *Amolopshimalayanus* from China in life. **A** Dorsal view; **B** ventral view; **C** left view; **D** right view.

Dorsal surface of the head and body green, some small black spots on dorsum; dorsal surface of limbs yellowish-green with some brown crossbars; lateral surface of head light green, a black stripe below canthus rostralis from snout tip across eyes to supratympanic fold; upper lip light yellowish-green with irregular brown spots; lateral surface of body light green, a large brown spot just behind supratympanic fold on each side; ventral surface of head light yellow, lower lip yellowish-brown; ventral surface of light greenish-yellow; ventral surface of limbs yellowish-brown (Fig. 2).

#### Ecological notes

**Figure 3.**
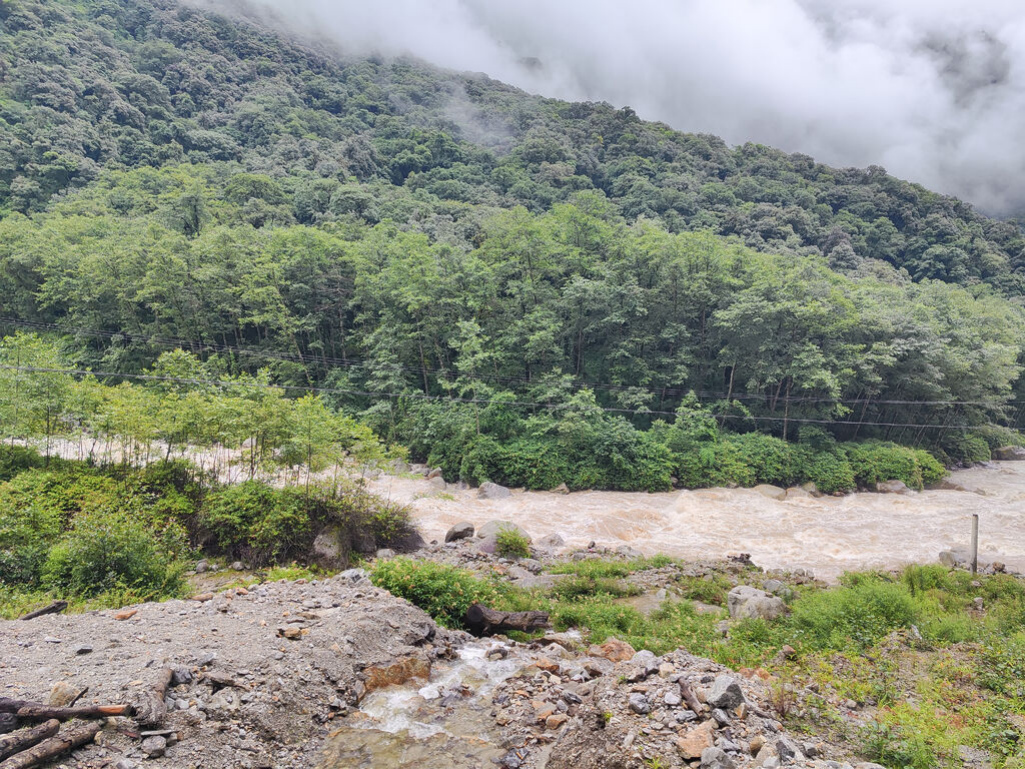
Habitat of the specimen of *Amolopshimalayanus* collected in China.

This specimen was found at night in the shrubland near a river on a leaf of an herbaceous plant (Fig. 3). Other amphibian species found in sympatry include *Nanoranablanfordii* (Boulenger, 1882), *N.liebigii* (Günther, 1860), *Raorchestesyadongensis* Zhang, Shu, Liu, Dong & Guo, 2022 and *Xenophryspangdaensis* Shu, Li, Wu, Liu, He, Li, Zhang & Guo, 2023.

#### Recommended common name

We suggest 喜山湍蛙 (Pinyin: xǐ shān tuān wā) as the Chinese name of this species.

## Analysis

**Figure 4.**
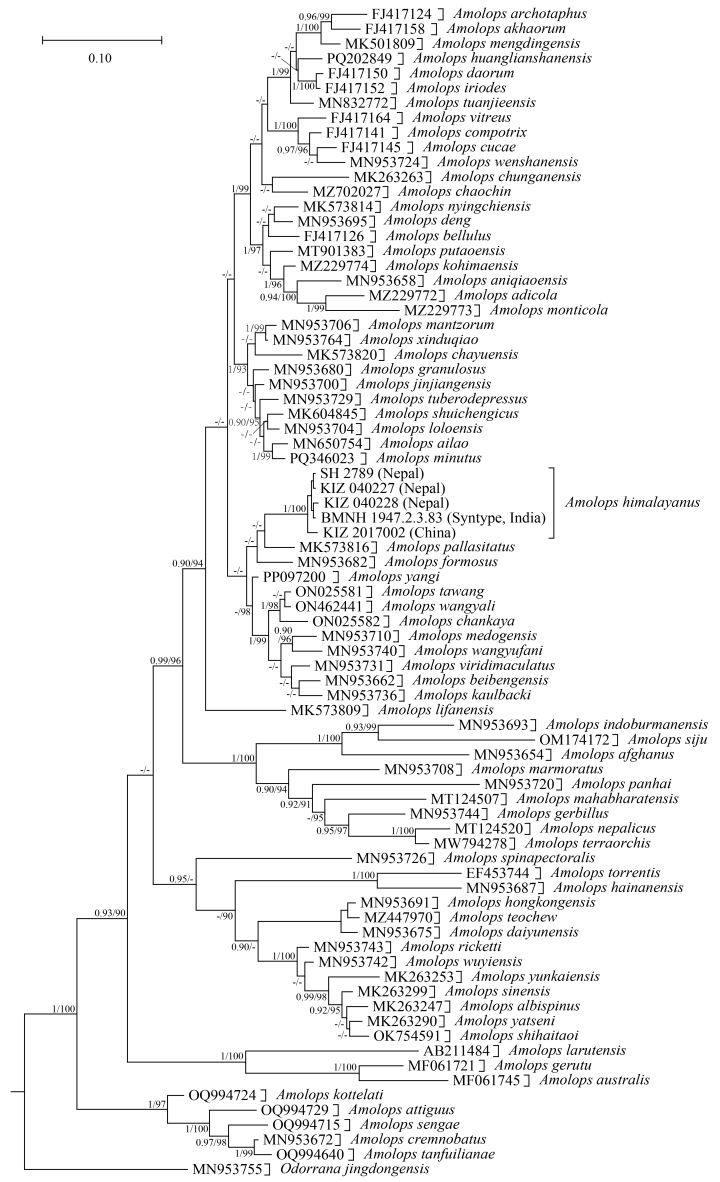
Bayesian phylogenetic tree based on 16S sequences. The numbers after and behind the “/” indicate the Bayesian posterior probabilities and Maximum Likelihood ultrafast bootstrap values (> 0.90/90), respectively.

Bayesian Inference and Maximum Likelihood analysis obtained similar results. The sequence of the specimen from Yadong, Xizang, China, clustered with the sequences of the syntype (BMNH1947.2.3.83) and other specimens of *Amolopshimalayanus* from Nepal with strong support (Fig. 4). The genetic distance (uncorrected p-distance) between the sequence of the specimen from Yadong and the sequence of the syntype (BMNH1947.2.3.83) of *A.himalayanus* was only 0.7% and the genetic distances (uncorrected p-distance) between the sequence of the specimen from Yadong and the sequences of the specimens of *A.himalayanus* from Nepal ranged from 0.8% to 0.9% (Table 3).

## Discussion

In some species of *Amolops*, especially in the *A.viridimaculatus* group, the body colouration of juveniles usually differs significantly from that of adults (Mahony et al. 2022). Therefore, the juvenile specimen we collected from Xizang cannot be accurately identified by morphology. However, phylogenetic analysis strongly supported that this specimen belongs to *A.himalayanus*, with a genetic distance of only 0.7% between it and the syntype (BMNH1947.2.3.83) of *A.himalayanus* in 16S gene sequences. Thus, we confirmed that *A.himalayanus* is distributed in China.

*Amolopshimalayanus* has rarely been reported since it was described more than a hundred years ago. Previously, this species was recorded in Nepal and Bhutan and the photos in life and breeding ecology of this species have been reported (Nidup et al. 2016, Limbu et al. 2020). Subsequently, Mahony et al. (2022) pointed out that the species previously recorded as *A.himalayanus* in Bhutan actually belonged to *Amolopswangyali* Mahony, Nidup, Streicher, Teeling & Kamei, 2022 and removed *A.himalayanus* from Bhutan's herpetofauna. In addition, Mahony et al. (2022) confirmed the distribution of *A.himalayanus* in Nepal based on genetic data, but did not provide any morphological data or photos of *A.himalayanus*. Although there are some photos of alleged *A.himalayanu* available on the website iNaturalist (https://www.inaturalist.org/), even some of them having been taken from the type locality of *A.himalayanu*, they have not undergone strict identification. Therefore, so far, no reliable photos of *A.himalayanus* in life have been formally reported. Herein, we record *A.himalayanus* from China and provide photos of this species in life for the first time, based on a genetically confirmed specimen, even though it is a juvenile.

**Figure 5.**
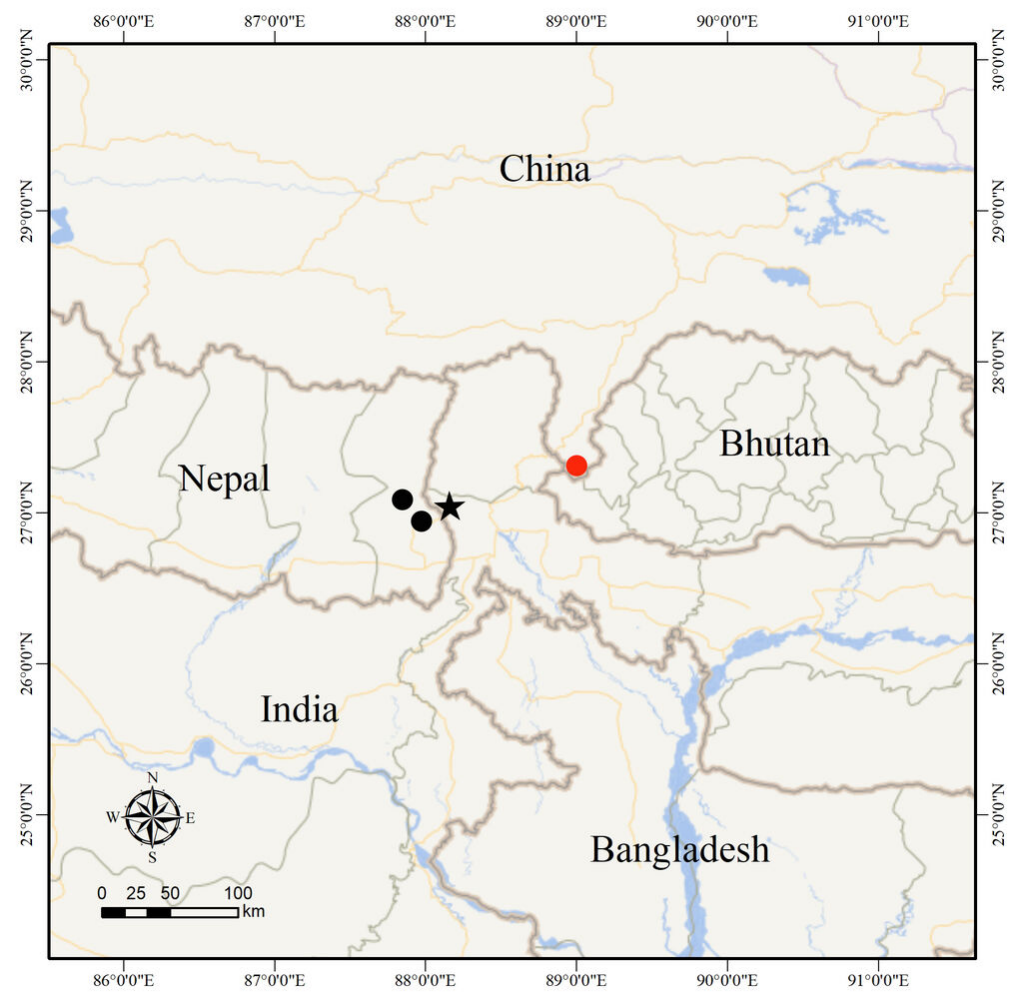
Map showing the type locality of *Amolopshimalayanus* in Darjeeling, India (black star), the confirmed distributions in Ilam and Mechi, Nepal (black dots) and the new collection site from Yadong, Xizang, China (red dot).

Previously, *Amolopshimalayanus* was confirmed to be distributed in Darjeeling, India, as well as in Ilam and Mechi, Nepal. This study recorded for the first time the distribution of this species in China and the new collection site extended the distribution range of this species to the northeast by approximately 80 km (Fig. 5). Unfortunately, we only collected one specimen of this species and it is a juvenile. Future field surveys in this area should be strengthened to understand the population status of this species in China.

## Supplementary Material

XML Treatment for Amolops himalayanus

## Figures and Tables

**Table 1.** Samples used for the phylogenetic analyses in this study.

Species	Voucher	Locality	Accession
*Amolopsadicola*	BNHS 6121	Upper Siang, Arunachal Pradesh, India	MZ229772
*Amolopsafghanus*	KIZ 048431	Husa, Yunnan, China	MN953654
*Amolopsailao*	GXNU YU000004	Xiping, Yunnan, China	MN650754
*Amolopsakhaorum*	FMNH 271355	Vieng Phou Kha, Luang Namtha, Laos	FJ417158
*Amolopsalbispinus*	SYS a003452	Shenzhen, Guangdong, China	MK263247
*Amolopsaniqiaoensis*	KIZ 011136	Xizang, China	MN953658
*Amolopsarchotaphus*	CUMZ A 2000.62	Doi Inthanon, Chiang Mai, Thailand:	FJ417124
*Amolopsattiguus*	NCSM 79166	Anh Son, Nghe An, Vietnam	OQ994729
*Amolopsaustralis*	LSUHC 7673	Endau-Rompin, Peta, Malaysia	MF061745
*Amolopsbeibengensis*	KIZ 016397	Medog, Xizang, China	MN953662
*Amolopsbellulus*	CAS 233986	Tengchong, Yunnan, China	FJ417126
*Amolopschankaya*	V/A/NERC/ZSI/1771	West Kameng, Arunachal Pradesh, India	ON025582
*Amolopschaochin*	CIB 116971	Chongzhou, Sichuan, China	MZ702027
*Amolopschayuensis*	SYS a007509	Baxoi, Xizang, China	MK573820
*Amolopschunganensis*	SYS a004212	Jinggangshan, Jiangxi, China	MK263263
*Amolopscompotrix*	FMNH 256500	Nakai, Khammouan, Laos	FJ417141
*Amolopscremnobatus*	KIZ 011621	Puhu National Reserve, Thanhhoa, Vietnam	MN953672
*Amolopscucae*	AMNH 168729	Van Ban, Lao Cai, Vietnam	FJ417145
*Amolopsdaiyunensis*	KIZ 08991	Daiyunshan, Fujian, China	MN953675
*Amolopsdaorum*	ROM 38501	Lao Cai, Sa Pa, Vietnam	FJ417150
*Amolopsdeng*	KIZ 014116	Zayü, Xizang, China	MN953695
*Amolopsformosus*	KIZ 012533	Gyirong, Xizang, China	MN953682
*Amolopsgerbillus*	KIZ 014086	Medog, Xizang, China	MN953744
*Amolopsgerutu*	RMB 21077	Gunung Tebu, Terengganu, Malaysia	MF061721
*Amolopsgranulosus*	SCUM 045823HX	Dayi, Sichuan, China	MN953680
*Amolopshainanensis*	SCUM 050243YJ	Wuzhishan, Hainan, China	MN953687
*Amolopshimalayanus*	BMNH 1947.2.3.83	Darjeeling, West Bengal, India	SAMN28238802
*Amolopshimalayanus*	KIZ 040227	Mabu, Ilam, Nepal	MN953713
*Amolopshimalayanus*	KIZ 040228	Maimajhuwa, Ilam, Nepal	MN953714
*Amolopshimalayanus*	SH 2789	Rakse village, Mechi, Nepal	MN953712
*Amolopshimalayanus*	KIZ 2017002	Yadong, Xizang, China	PV241793
*Amolopshongkongensis*	ROM 29014	Hong Kong, China	MN953691
*Amolopshuanglianshanensis*	KIZ 2023094	Lvchun, Yunnan, China	PQ202849
*Amolopsindoburmanensis*	CAS 233204	Haka, Chin, Myanmar	MN953693
*Amolopsiriodes*	AMNH 163926	Vi Xuyen, Ha Giang, Vietnam	FJ417152
*Amolopsjinjiangensis*	SCUM 050434CHX	Deqing, Yunnan, China	MN953700
*Amolopskaulbacki*	SCUM 050402CHX	Pianma, Yunnan, China	MN953736
*Amolopskohimaensis*	WIIADA 751	Kohima, Nagaland, India	MZ229774
*Amolopskottelati*	NCSM 79617	Thaphabhat, Bolikhamxay, Laos	OQ994724
*Amolopslarutensis*	KUHE 15488	Perak, Malaysia	AB211484
*Amolopslifanensis*	SYS a005374	Lixian, Sichuan, China	MK573809
*Amolopsloloensis*	SCUM 045806HX	Xichang, Sichuan, China	MN953704
*Amolopsmahabharatensis*	CDZMTU 0110	Chitwan, Bagmati, Nepal	MT124507
*Amolopsmantzorum*	SCUM 045817HX	Wolong, Sichuan, China	MN953706
*Amolopsmarmoratus*	KIZ 013411	Huai Hea, Chiang Mai, Thailand	MN953708
*Amolopsmedogensis*	SYNU 04II6216	Medog, Xizang, China	MN953710
*Amolopsmengdingensis*	KIZ 20160266	Mengding, Yunnan, China	MK501809
*Amolopsminutus*	IEBR Amolops5142	Tam Duong, Lai Chau, Vietnam	PQ346023
*Amolopsmonticola*	WIIADA 544	Tarku, Sikkim, India	MZ229773
*Amolopsnepalicus*	CDZMTU 0148	Lamatar, Taplejung, Nepal	MT124520
*Amolopsnyingchiensis*	SYS a006679	Medog, Xizang, China	MK573814
*Amolopspallasitatus*	SYNU 1507034	Dinggye, Xizang, China	MK573816
*Amolopspanhai*	FMNH 268355	Huay Yang, Prachuap Khiri Khan, Thailand	MN953720
*Amolopsputaoensis*	GXNU W011	Putao, Kachin, Myanmar	MT901383
*Amolopsricketti*	HDSK 0043	Wuyishan, Fujian, China	MN953743
*Amolopssengae*	FMNH 258376	Kasi, Vientiane, Laos	OQ994715
*Amolopsshihaitaoi*	GXNU YU000353	Hekou, Yunnan, China	OK754591
*Amolopsshuichengicus*	SYS a004956	Shuicheng, Guizhou, China	MK604845
*Amolopssiju*	D414	Siju, Meghalaya, India	OM174172
*Amolopssinensis*	SYS a007107	Yingde, Guangdong, China	MK263299
*Amolopsspinapectoralis*	ROM 37375	Ngoc Linh, Kon Tum, Vietnam	MN953726
*Amolopstanfuilianae*	AMS R 171526	Con Cuong, Nghe An, Vietnam	OQ994640
*Amolopstawang*	V/A/NERC/ZSI/1772	Tawang, Arunachal Pradesh, India	ON025581
*Amolopsteochew*	SYS a008705	Chaozhou, Guangdong, China	MZ447970
*Amolopsterraorchis*	Amolops_331	Arunachal Pradesh, India	MW794278
*Amolopstorrentis*	SCUM 050253YJ	Hainan, China	EF453744
*Amolopstuanjieensis*	GXNU YU 110003	Gengma, Yunnan, China	MN832772
*Amolopstuberodepressus*	SCUM 050433CHX	Jingdong, Yunnan, China	MN953729
*Amolopsviridimaculatus*	KIZ 048487	Tengchong, Yunnan, China	MN953731
*Amolopsvitreus*	FMNH 258187	Phongsaly, Phongsaly, Laos	FJ417164
*Amolopswangyali*	SCZM 2019.07.18.1	Bodidrang Chhu, Trashigang, Bhutan	ON462441
*Amolopswangyufani*	KIZ 014067	Zayü, Xizang, China	MN953740
*Amolopswenshanensis*	KIZ 021425	Xichou, Yunnan, China	MN953724
*Amolopswuyiensis*	HDSK 0042	Wuyishan, Fujian, China	MN953742
*Amolopsxinduqiao*	KIZ 041127	Kangding, Sichuan, China	MN953764
*Amolopsyangi*	KIZ 050788	Fugong, Yunnan, China	PP097200
*Amolopsyatseni*	SYS a006807	Zhongshan, Guangdong, China	MK263290
*Amolopsyunkaiensis*	SYS a003979	Yangchun, Guangdong, China	MK263253
*Odorranajingdongensis*	KIZ 46977	Jingdong, Yunnan, China	MN953755

**Table 2.** Measurements (in mm) of the specimen of *Amolopshimalayanus* from China (for abbreviations, see Material and Methods).

	KIZ 2017002		KIZ 2017002
SVL	22.9	ED	3.7
HL	8.6	TD	1.0
HW	8.0	LAHL	12.6
SL	3.7	HND	8.2
INS	3.2	LAD	1.7
IOS	3.0	FEM	12.4
NED	2.0	TIB	12.8
UEW	2.1	FTL	11.4

**Table 3.** Uncorrected pairwise genetic distances (%) between specimens of *Amolopshimalayanus*, based on 16S sequences.

	1	2	3	4
1 BMNH 1947.2.3.83 (Syntype, India)				
2 SH 2789 (Nepal)	0			
3 KIZ 040227 (Nepal)	0	0		
4 KIZ 040228 (Nepal)	0.2	0.2	0.2	
5 KIZ 2017002 (China)	0.7	0.8	0.8	0.9
